# Applied behavioral and decision sciences in support of US FDA’s drug regulatory mission

**DOI:** 10.1073/pnas.2525995123

**Published:** 2026-07-06

**Authors:** Sara L. Eggers, Tamar Krishnamurti, Baruch Fischhoff

**Affiliations:** ^a^Independent Scholar, Takoma Park, MD 20912; ^b^ Division of General Internal Medicine, University of Pittsburgh School of Medicine, Pittsburgh, PA 15213; ^c^ Department of Engineering and Public Policy and Carnegie Mellon Institute for Strategy and Technology, Carnegie Mellon University, Pittsburgh, PA 15213

**Keywords:** regulatory decision making, benefit–risk assessment, patient-focus, decision analysis, systems modeling

## Abstract

The US Food and Drug Administration (FDA) makes and communicates decisions that directly affect the health choices and well-being of the US public. Behavioral and decision scientists have long supported FDA’s pharmaceutical (medical drug) regulatory and public health mission, working alongside clinical and pharmaceutical scientists. This Perspective describes four interrelated cases illustrating these collaborations: a) creating the Benefit–Risk Framework to guide regulatory decisions regarding new drug approvals; b) launching an internal decision support service to facilitate specific regulatory decisions; c) implementing the Patient-Focused Drug Development initiative to strengthen the use of patient input to inform regulatory decision making; and d) developing FDA SOURCE, a dynamic systems simulation model to assess potential strategies to address the US opioid overdose crisis. Key to the success of these efforts has been their behavioral and decision science foundations, strategically implemented by an expert team dedicated to fostering trusted collaborations with internal and external partners. These cases offer a model for other agencies facing complex decisions of public impact.

The US Food and Drug Administration (FDA) is charged with ensuring the efficacy, safety, and quality of the US medical supply. The scope of its jurisdiction is immense, including pharmaceuticals (medical drugs), blood products and biologics, gene and cell therapies, and medical devices. Its regulatory authority includes oversight of human studies of investigational products, approval of new products, and monitoring of marketed products. FDA also monitors products that do not require regulatory approval, such as compounded pharmaceuticals (i.e., individually made-to-order by a pharmacist or physician) and homeopathic remedies (1).

FDA’s mission is to make regulatory decisions on behalf of the US public. These decisions are made in its Centers and Offices, with specialized authorities and expertise. The Center for Drug Evaluation and Research (CDER), the focus of this article, makes several hundred major decisions each year about new drug marketing approvals (2), expanded approved uses for already marketed products (3), and major safety labeling or other risk management requirements for marketed products (4, 5). CDER makes thousands of more incremental decisions regarding clinical trial design, product labeling, manufacturing specifications, advertising compliance, and more. FDA is also charged with communicating authoritative information to aid health decisions, including educational campaigns (e.g., ref. 6), consumer and healthcare provider alerts (e.g., ref. 7), and regulatory decision documentation (e.g., ref. 8).

Many of FDA’s scientific decisions and communications are relatively routine and straightforward. Some decisions, though, are as complicated as any faced by a regulatory agency. When evaluating a new drug, FDA reviews extensive study protocols, scientific evidence, and manufacturing plans, as well as input from patients and its expert advisory committees. It must consider uncertainties stemming from poorly understood disease processes, limited or imperfect study data, complex medical use settings, and often limited understanding of patients’ perspectives. In drawing its conclusions, FDA must balance the needs and desires of individual patients (e.g., varying in disease severity and risk tolerances) with its responsibility to the patient population as a whole (e.g., requiring judgments about intolerable risks and uncertainties) and to public health more broadly (e.g., considering disease transmission, substance misuse) (9). All of this work must be done within regulatory constraints, mandated by the US Congress, regarding what factors can be considered, what requirements can be dictated, and what information can be shared.

The quality of FDA’s decisions and communications depends on its understanding of how people make decisions and what information they need to support them (10). Starting in the mid-2000‘s, FDA strategically strengthened its social and behavioral science capabilities (11) to work alongside its long-standing medical and pharmaceutical expertise. Principles and practices of decision science, behavioral epidemiology, systems modeling, risk communication, and implementation science have all become parts of FDA‘s formal analysis, research, participatory engagement, and practical interventions.

This Perspective illustrates these collaborations with four interrelated cases showing practical applications of behavioral and decision science that support FDA’s regulatory mission to assess the benefits and risks of pharmaceuticals. These cases represent an evolutionary arc. The first, creating the Benefit–Risk Framework for New Drug Review, revealed specific needs that were addressed in the subsequent three, including internal decision support (Case 2), strengthened patient input (Case 3), and advanced systems-thinking approaches (Case 4). These cases originated in (what is currently called) CDER’s Office of Strategic Programs, with the mission to elevate strategic and analytic approaches to support regulatory and operational functions, and expertise including decision science, data analytics, economics, and program implementation. For each case, we highlight its scientific grounding, execution, and accomplishments. While we focus on work in CDER, other FDA medical product centers have followed similar paths.

## Case 1. The Benefit–Risk Framework for New Drug Review

For a drug to be approved for marketing in the United States, FDA must determine that its benefits (in terms of diagnosing, preventing, treating, or curing a medical condition) outweigh its risks of short- and long-term adverse events (9). FDA has been dedicated to this role since its mandate by the 1938 Food, Drugs, and Cosmetics Act. Over time, the increased complexity of medical product development and evaluation has amplified the need for consistent, transparent, and accessible documentation of FDA’s assessments, prompting the Institute of Medicine to recommend in 2007 that FDA “develop and continually improve a systematic approach to risk–benefit analysis for use throughout the FDA in the preapproval and postapproval settings” [(12), p. 125].

FDA responded to this recommendation by creating the Benefit–Risk Framework for New Drug Review as a structured approach for conducting drug benefit–risk assessments and a vehicle for explaining the basis for FDA’s drug approval regulatory decisions (13). FDA committed to implementing the Framework in the 2012 reauthorization of the Prescription Drug User Free Act (PDUFA V) (14). Led by internal staff in strategic planning and analysis and supported by external experts in behavioral and decision science, FDA undertook an iterative, consultative approach to developing the Framework and incorporating it within FDA’s decision-making processes. Implementation included document templates, staff training, desk-side support, internal oversight, and third-party evaluations (15). FDA further issued guidance to clarify to the public its benefit–risk assessment approach and inputs (9).

**Fig. 1.**
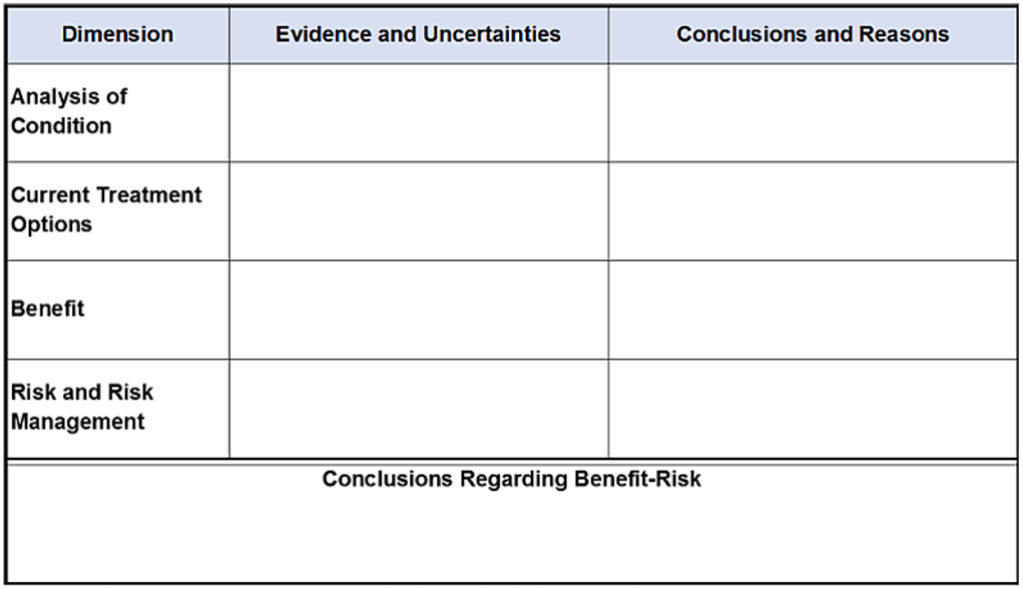
The FDA Benefit–Risk Framework, template shell [Image credit: Reprinted from ref. (9), p. 8].

The defining feature of the Benefit–Risk Framework is its simplicity. As shown by Fig. 1, the Framework shell is applicable to any drug benefit–risk assessment pre- or postapproval. FDA staff consider the table elements throughout their comprehensive review of a drug application and finalize the cell entries at its conclusion. The rows reflect the dimensions of benefit–risk decision making. *Analysis of the Condition* establishes the disease burden and patients’ medical needs. *Current Treatment Options* summarizes how well those needs are currently being met. The next two rows focus on the product under evaluation, summarizing its expected *Benefits* and *Risks,* including measures to manage its risks. The Framework’s design reflects decision science principles, with its rows specifying critical decision-making dimensions, and its columns distinguishing scientific facts (*Evidence and Uncertainties*) and value-laden judgments (*Conclusions and Reasons*). The final element, *Conclusions Regarding Benefit-Risk*, provides the integrated judgment of benefits versus risks, considering the therapeutic context.

The Benefit–Risk Framework is grounded in the principles that guide decision analysis (16): Define what matters most (benefits and risks), articulate the options for patients (the proposed treatment, alternative treatments, no treatment), predict the outcomes with their uncertainties, consider the tradeoffs, and identify what more is needed to reduce risk and uncertainty to acceptable levels. The Framework, supported by guiding prompts, encourages broad consideration of these critical elements. Respecting the case-specific and deeply nuanced judgments that are necessarily involved, the Benefit–Risk Framework is fundamentally a qualitative decision analysis of tradeoffs and uncertainties. More advanced model-based (e.g., multicriteria) decision analyses can be incorporated when meeting a specific need revealed by the qualitative analysis (9, 15).

Completed Benefit–Risk Frameworks are found in the Executive Summary of FDA CDER’s integrated review documents posted for approved drugs (8). Illustrative examples include pacritinib [(17), p. 5)], a treatment for a rare blood disorder, and odevixibat [(18), p. 5], a treatment for pruritus (severe itching) associated with a rare liver disease. Predecisional Frameworks may also be included in the FDA briefing document prepared for external Advisory Committee meetings that may occur in advance of a regulatory decision to summarize important benefit–risk considerations (e.g., ref. 19, p. 43). As a communication tool, each Framework provides insight into a specific decision. Together, they show how FDA approaches its decisions (20).

FDA’s Benefit–Risk Framework and the associated guidance are recognized among industry, patient groups, and regulators as a model for pharmaceutical benefit–risk assessment and communication (21). FDA CDER has similarly advanced decision principles with a complementary framing tool, in the form of a logic model, to structure decisions about risk management measures that may be necessary to ensure that a drug’s benefits outweigh its risks in real-world settings, where issues of risk mitigation effectiveness and impacts on treatment access and patient burden must be considered (22, 23).

An opportunity for greater use of the FDA Benefit–Risk Framework is in the postmarket setting. FDA’s oversight extends beyond marketing approval, recognizing that benefit–risk profiles can change as new information accrues after the drug is on the market (9). The Benefit–Risk Framework structure applies here too, in making explicit the evolving therapeutic context, evidence, uncertainties, and tradeoffs. It can provide even greater value as the foundation for structured assessments throughout the product lifecycle, from development through marketing, providing efficiency, continuity, and transparency to an iterative assessment over time.

## Case 2. Regulatory Decision Support

The success of the Benefit–Risk Framework prompted FDA CDER to further expand its decision science capabilities. By 2020, the “Benefit–Risk Team” evolved to the Decision Support and Analysis Staff (DSAS), a small team of operations research analysts and social scientists charged with leveraging decision science to structure, inform, and communicate the Center’s drug regulatory decision making (24). This included more challenging benefit–risk assessments and other decisions involving multifaceted regulatory goals (e.g., pertaining to drugs with potential for misuse, over-the-counter medications, and medication use during pregnancy). DSAS resided outside any regulatory review function (e.g., clinical, statistics, legal) and the executive decision chain, providing third-party support focused on decision-making processes (25). DSAS offered behavioral and decision science expertise and by-request internal consultative services. Its work included facilitation, analysis, and research, falling into three basic categories, summarized below in order of increasing effort, with simplified examples.

### Decision Structuring and Qualitative Analysis.

These applications involve a facilitated process (26) and supporting analyses to help a team frame its decision context, objectives, options, uncertain outcomes, and tradeoffs. Such qualitative work was often sufficient to meet the client’s needs.

Example: In 2020, FDA faced a regulatory decision about modifying the labeling of montelukast, a medication to treat asthma and allergic rhinitis and commonly used in children (27). The need for a decision was prompted by postmarketing concerns about potential risks of neuropsychiatric adverse events. The regulatory options ranged from requiring further study (among the least restrictive actions) to requiring a “boxed warning” (28) or narrowing the product’s approved use (among the most restrictive actions). The choice was complicated by scientific uncertainty about the relationship between the drug and the adverse events; a therapeutic context that included both allergic rhinitis (a bothersome condition with several treatment options) and asthma (a more serious condition with fewer treatment options); and complex regulatory factors such as decisional precedent. DSAS facilitated a qualitative decision analysis (29) helping the review team articulate its regulatory objectives, which included minimizing inappropriate off-label use, maintaining treatment availability to patients for whom the drug’s benefits outweighed risks, and ensuring regulatory consistency. They then helped the review team assess the likely impact of each regulatory option on those objectives and the potential for unintended consequences. These deliberative processes informed FDA’s eventual decision to narrow montelukast‘s approved use for allergic rhinitis and to add a boxed warning in labeling.

### Formal (Quantitative) Decision Analysis.

These analyses expand qualitative benefit–risk assessment by quantifying judgments of the relative importance of different benefit and risk outcomes (30). These importance weights can be derived from patient preference studies, published health utility metrics, or elicited regulatory preferences (31). CDER’s internal use of these methods has shown emerging value for some review decisions in certain therapeutic areas, such as cardiovascular and kidney diseases.

Example: In 2019, FDA CDER reviewed an application to expand approval for the antiplatelet drug ticagrelor to include primary prevention of cardiovascular (CV) events, the first use of its kind for an antiplatelet drug. FDA’s qualitative benefit–risk assessment, using the clinical trial data, suggested that the drug causes greater harm outcomes (e.g., major bleeding events in the brain and elsewhere) than the disease outcomes it prevents (heart attacks and strokes). The review team believed a more nuanced accounting of the clinical importance of the various outcomes was needed. DSAS led a multicriteria decision analysis and elicited reviewers’ subjective weighting of the clinical importance of the key outcomes (32). This quantitative analysis, along with its deliberative process, led the review team to reexamine some key uncertainties and ultimately supported a more confident approval decision. The analysis received internal accolades in 2022 (33) for advancing social and behavioral sciences.

### Generating Decision-Relevant Information.

At times, decision structuring reveals specific missing information that could inform the evaluation of decision options. For such cases, Decision Support contributed decision-focused research and analyses including expert elicitation, multiparty facilitation, risk modeling, and perceptions research with patient and healthcare providers.

Example: In 2023, FDA approved the first over-the-counter (OTC) oral contraceptive (34). This approval decision was challenged by study design and data flaws that limited FDA’s ability to assess consumers’ adherence to the medication. Without adherence information, regulators can only evaluate the proposed product’s safety and effectiveness in intention-to-treat terms (35). To support the review team’s assessment of this uncertainty in the benefit–risk context, Decision Support analyzed study participants’ reported prior contraceptive methods (including none) to approximate a status quo population-level contraception failure rate, to serve as a proxy threshold by which to compare the proposed product’s efficacy. Results from this analysis reassured the signatory authority (34) that, even with the uncertainties about adherence, the target population would expect greater benefits (i.e., fewer unintended pregnancies) with the product available than without it.

## Case 3. Patient-focused Drug Development

Patients with direct experience with an illness and its treatment can offer important insight into drug evaluation, including benefit–risk assessment. Historically, patients and caregivers had little structured input to FDA drug review beyond the standard right to provide public comment at Advisory Committee meetings (36). Recognizing the value of patients’ input, FDA also committed in 2012 PDUFA V to a series of disease-specific public meetings dedicated to hearing patients’ perspectives on their condition and current treatments (14). These meetings launched FDA’s Patient-Focused Drug Development (PFDD) initiative (37), which has played a seminal role in advancing methods to generate patient input, in the United States and beyond (38).

The PFDD meetings were created and initially conducted by the team that led the Benefit–Risk Framework, aligning the two initiatives. Drawing on behavioral science principles (39, 40) which included seeking input from patient stakeholders (41), PFDD meetings were designed to foster accessible, meaningful, and mutually respectful engagement. They have many features that differ from standard FDA public meetings. They are structured yet conversational, with only patients and caregivers providing input during the main session. The meeting dialogue centers on a set of open-ended questions (Table 1) regarding patients’ daily experience, treatment needs, and perspectives on currently available treatments (i.e., the top two rows of the Benefit–Risk Framework). FDA facilitators use guiding prompts and polling to enable all participants to present their experiences. A panel of FDA experts can ask follow-up questions relevant to their regulatory work. To increase accessibility, FDA enables virtual meeting participation and encourages submission of written comments to the *Federal Register* docket for each meeting.

**Table 1.** Standard meeting questions tailored for each PFDD meeting

**Topic 1. Health effects and daily impacts that matter most to patients**
Of all the symptoms or disease manifestations you experience because of your condition, which 1 to 3 have the most significant impact on your life? Are there specific activities that are important to you but that you cannot do at all or as fully as you would like because of your condition? How do your symptoms and their negative impacts affect your daily life on the best days? On the worst days? How has your condition changed over time? What worries you most about your condition?
**Topic 2. Patients’ perspectives on current approaches to treatment** What are you currently doing to help treat your condition or its symptoms? How has your treatment regimen changed over time? How well does your current treatment regimen manage your condition? What are the most significant downsides to your current treatments and how do they affect your daily life? What specific things would you look for in an ideal treatment? What factors do you take into account when making decisions about selecting a course of treatment?

After each PFDD meeting, FDA produces a “Voice of the Patient” Report, thematically summarizing the meeting transcript, meeting web comments, and submitted docket comments. Care is taken to reflect participants’ words and terms, while acknowledging that the report may not capture the full breadth of patient experiences and views.

The first PFDD meeting, in April 2013, was for chronic fatigue syndrome/myalgic encephalomyelitis, a complex, serious condition with diagnostic challenges and no approved treatments. Input from this meeting directly informed a 2014 FDA Guidance on developing drug treatments (42), as well as a 2015 Institute of Medicine report on disease diagnostic criteria (43). FDA has led 30 meetings since, on conditions including sickle-cell disease, alopecia areata, psoriasis, chronic pain, and opioid use disorder. In response to public enthusiasm, FDA has also franchised its approach, enabling patient groups to organize and lead their own meetings. More than 100 meetings have been conducted through this external pathway. FDA hosts the reports of its meetings and those conducted by external parties in a repository (44) for the benefit of drug developers, researchers, patients, and advocates. Analyzing the portfolio of meetings can reveal concerns that cut across chronic conditions, including physical, mental, emotional, social, and financial impacts, as well as broader issues regarding treatment benefit, burden, and access (45).

Building on the success of this initiative, FDA CDER created a Patient-Focused Drug Development Staff charged with further advancing programs and policies that facilitate the incorporation of patient-derived input in drug development and evaluation.

## Case 4. Opioid System Modeling

Opioid medications present a special challenge for benefit–risk assessment (46). While they can provide meaningful benefits to patients who require them to manage severe pain, they impose direct risks to patients and indirect risks to society. Problematic opioid use and opioid overdose continue to be among the most significant US public health challenges. As the authority responsible for regulating opioid medications, FDA plays an important role in mitigating the crisis. Its public health priorities include decreasing unnecessary exposure to prescription opioids, protecting against illicit opioids, mitigating harms from overdose, and supporting individuals who have opioid use disorder (OUD) (47).

Assessing the impact of opioid regulatory and policy interventions is complicated by the ever-changing opioids landscape involving interdependent physiological, behavioral, social, and geopolitical forces. For example, “abuse-deterrent formulations” intended to deter nonmedical use of prescription opioids can unintentionally drive people who seek opioid effects to switch to more dangerous illicit opioids, such as fentanyl (48). Recognizing this complexity, the National Academies of Science, Engineering and Medicine (NASEM) recommended in 2017 that FDA adopt a systems approach to incorporate broader public health complexities when evaluating the expected benefits and risks of potential regulatory actions and policies (49).

FDA responded by creating a dynamic systems model of the opioid overdose crisis (50). Its goals were to 1) identify interventions with high-impact potential, 2) assess the intended and unintended consequences of potential policies, and 3) identify critical systems-level uncertainties for future research. The intent was to set the model within a policy analysis service that could meet regulatory needs. The initiative was led by an internal FDA team representing decision science, system modeling, data analysis, and program implementation expertise. The team partnered with academic researchers who created the model, advisory contractors who supported scientific review and service implementation, and internal FDA experts in opioids and regulatory policy who provided data and established the regulatory needs.

The initiative produced FDA SOURCE (Simulation of Opioid Use, Response, Consequences, and Effects), a national-level dynamic systems simulation model, calibrated to 22 y of historical data (1999 to 2020) from multiple sources (51). The model, supporting data, and documentation are publicly available (https://github.com/FDA/SOURCE). SOURCE models the movement of US persons age 12 and older through the stages of opioid misuse, use disorder, treatment, and remission, tracking key public health outcomes and producing estimates of the prevalence of opioid use disorder and opioid overdose deaths. As a tenet of dynamic systems modeling, SOURCE incorporates feedback mechanisms, sensitive to changes in social influences, perceived risks, and OUD treatment availability. Model simulations can project potential future trajectories of opioid outcomes conditional on possible future policies, including no change at all (i.e., baseline).

Lim et al. (51) presents SOURCE baseline projections (i.e., assuming no change in policies) for 2020 to 2032 for key metrics, including opioid misuse initiation, opioid use disorder, and overdose deaths. A striking initial finding was the projection that opioid overdose deaths in the United States were nearing a peak and would begin declining within a few years. This result was contrary to other modeling at the time, as well as the prevailing concern given the sharp rise in US overdose deaths that occurred during and immediately following the COVID pandemic. SOURCE, with its novel social feedback, attributed this peak and decline in part to accelerating growth in perceptions of the increasingly deadly effects of opioids (e.g., with the proliferation of fentanyl) resulting in reduced opioid use. As SOURCE had qualitatively projected, CDC’s opioid-related death count plateaued in 2023 and declined by 24% between Sept 2023 and Sept 2024 (52). While this decline in no way diminishes the tragedy of opioid-related deaths, it offers some hope for turning the tide. The finding also illustrates the insights that systems modeling efforts can bring to policy assessments.

**Fig. 2.**
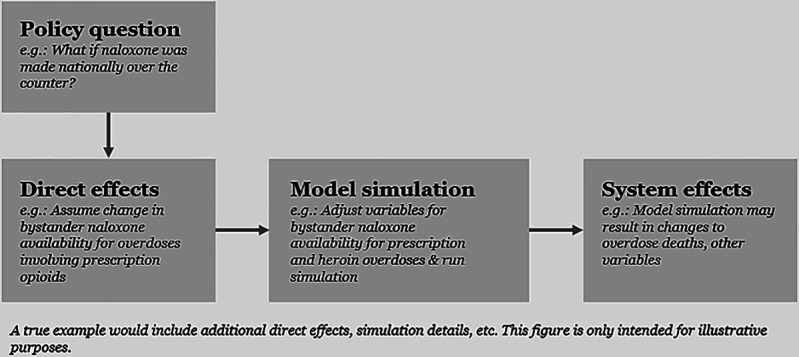
FDA SOURCE workflow, translating policy questions into model analysis [Image credit: Reprinted from ref. (50), p. 13].

SOURCE has been applied to explore potential federal-level policies, following the basic workflow in Fig. 2. For example, Stringfellow et al. (53) projected that the largest and fastest impacts on reducing opioid overdose death could come strategies that reduce exposure to highly potent fentanyl (e.g., using test strips to detect fentanyl-contaminated products) and increasing the availability of the overdose reversal product naloxone, although both strategies would slightly increase OUD prevalence. In contrast, increasing “recovery supports” that help people stay in OUD remission, would reduce both opioid overdose deaths and the prevalence of OUD.

The insights generated by SOURCE, coupled with decision support, have informed FDA’s internal assessments and decisions, for example, to authorize over-the-counter naloxone (54). Complementary qualitative systems modeling has also informed discussion of complex opioid policy topics such as regulating abuse deterrent formulations (55), high-dosage opioid pills (56), and medication to treat OUD (57). The opioids systems modeling initiative has been recognized within FDA (58) and externally (59) as exemplary of applying systems science to support its regulatory mission.

## Discussion

The four cases highlighted in this Perspective demonstrate how FDA has embedded behavioral and decision science research, principles, and practices in direct support of its regulatory mission. These examples focused on pharmaceutical benefit–risk assessment. There are many other examples, including benefit–risk analysis to support review of vaccines, blood products, and medical devices (e.g., ref. 60); behavioral research portfolios supporting FDA’s oversight of prescription drug promotion (61), over-the-counter medication, medication safety, and health equity (62); decision-analytic approaches to inform drug risk management (63); and leveraging external expertise through the FDA Risk Communication Advisory Committee to inform decision making and communications (64).

One key lesson from these cases is the need for synergistic collaboration, both internally and with external partners, including experts, researchers, and end-users. FDA needed these partners to ensure it had the best expertise, while those partners needed FDA support to ensure regulatory applicability. Collaboration success required strategic efforts to coordinate diverse teams, navigate operational complexities (e.g., funding, contracting, data acquisition) and balance the various parties’ objectives and constraints.

FDA’s ability to incorporate behavioral and decision sciences has depended not only on its own internal absorptive capacity but also on the availability of basic research relevant to its practical concerns. For example, research on numerical displays has shown how to communicate quantitative information (e.g., using well-defined numerical estimates, rather than vague verbal quantifiers) (65, 66). Research on visual displays has shown how to structure complex information (67, 68). Research on terminology has shown how well technical language communicates to nonexpert users (69). By creating sustainable, meaningful partnerships with experts, agencies can increase the chance that research is available when opportunities arise that need it.

A second lesson has been the need for patience and commitment in building trust. FDA reviewers need to be confident that the simple Benefit–Risk Framework can adequately capture their nuanced decisions (Case 1) and that decision analysis will not take away their decisional autonomy (Case 2). FDA’s leaders and patients need confidence that patient-focused meetings will use their time well and have a meaningful impact (Case 3). FDA’s opioids experts and policy makers need to trust that SOURCE is robust and transparent enough to be support consequential decisions (Case 4).

Perhaps the greatest insight from these cases is the importance of having a dedicated internal staff, with expertise in behavioral and decision science, deep familiarity with FDA’s regulatory context, and the strategic ability to formulate and execute programs. Meeting all these conditions, for example, ensured that FDA’s Benefit–Risk Framework was scientifically grounded, fit-for-purpose, and sustainable. Its design and implementation process provide lessons for any such team (70):

- Engage decision- makers, early and often.
- Understand and focus on the decision makers’ problem.
- Demonstrate how the tool will add value over the status quo, at an acceptable cost (or reduce costs with more efficient processes).
- Seek and value nuanced subject matter expertise.
- Respect the regulatory context and constraints.
- Scale to the needed level of complexity. Do what is needed and no more.
- Assess (and reassess) the boundaries of the decision makers’ comfort with new approaches.

Organizational changes at FDA in 2025 significantly altered the agency’s operational framework, with direct implications on FDA CDER’s behavioral and decision science expertise and capabilities. As of April 2025, FDA CDER’s Decision Support and Analysis Staff is no longer in service, nor is the Center’s risk communications research function. Nonetheless, their scientific and operational contributions have created lasting value for regulatory decisions, through the Benefit–Risk Framework, SOURCE and other tools, principles disseminated in training, and a history of worked examples. Given that legacy and its flexible, problem-solving nature, a decision support infrastructure could be readily recreated. We hope for the return of such an explicit function.

FDA’s adoption of behavioral and decision science may serve as a model for other regulatory agencies responsible for difficult decisions involving imperfect evidence, complex behaviors, and difficult tradeoffs. Creating those capabilities requires leadership champions, a dedicated internal team of behavioral and decision science experts committed to the agency’s mission, agile tools that can be adapted to diverse regulatory purposes, and trust-building at all levels. The benefits are behavioral and decision science insights tuned to regulatory needs, reducing burden on staff, and ultimately, producing clearer decisions.

## Data Availability

There are no data underlying this work.
